# Whole Genome Sequencing Analysis to Identify Candidate Genes Associated With the rib eye Muscle Area in Hu Sheep

**DOI:** 10.3389/fgene.2022.824742

**Published:** 2022-03-14

**Authors:** Yuan Zhao, Xiaoxue Zhang, Fadi Li, Deyin Zhang, Yukun Zhang, Xiaolong Li, Qizhi Song, Bubo Zhou, Liming Zhao, Jianghui Wang, Dan Xu, Jiangbo Cheng, Wenxin Li, Changchun Lin, Xiaobin Yang, Xiwen Zeng, Weimin Wang

**Affiliations:** ^1^ College of Animal Science and Technology, Gansu Agricultural University, Lanzhou, China; ^2^ The State Key Laboratory of Grassland Agro-ecosystems, College of Pastoral Agriculture Science and Technology, Lanzhou University, Lanzhou, China; ^3^ Engineering Laboratory of Sheep Breeding and Reproduction Biotechnology in Gansu Province, Minqin, China; ^4^ Linze County Animal Disease Prevention and Control Center of Gansu Province, Linze, China

**Keywords:** whole genome sequencing, case-control GWAS, genetic parameters, association analysis, sheep breeding

## Abstract

In sheep meat production, the rib eye area is an important index to evaluate carcass traits. However, conventional breeding programs have led to slow genetic progression in rib eye muscle area. Operationalizing molecular marker assisted breeding is an optimized breeding method that might improve this situation. Therefore, the present study used whole genome sequencing data to excavate candidate genes associated with the rib eye muscle. Male Hu lambs (*n* = 776) with pedigrees and 274 lambs with no pedigree were included. The genetic parameters of the rib eye area were estimated using a mixed linear mixed model. The rib eye area showed medium heritability (0.32 ± 0.13). Whole-genome sequencing of 40 large rib eye sheep [17.97 ± 1.14, (cm^2^)] and 40 small rib eye sheep [7.89 ± 0.79, (cm^2^)] was performed. Case-control genome-wide association studies and the fixation index identified candidate rib eye-associated genes. Seven single nucleotide polymorphisms (SNPs) in six genes (*ALS2*, *ST6GAL2*, *LOC105611989*, *PLXNA4*, *DPP6*, and *COL12A1*) were identified as candidates. The study population was expanded to 1050 lambs to perform KASPar genotyping on five SNPs, which demonstrated that SNPs in *LOC105611989*, *DPP6*, and *COL12A1* correlated significantly with the rib eye area, which could be used as genetic markers for molecular breeding of the rib eye area. The results provided genetic parameters estimated on the rib eye area and information for breeding based on carcass traits in Hu sheep.

## Introduction

Improvement of carcass traits is very important for farmers and the sheep industry, because the commercial value of a sheep carcass is determined by a range of different aspects, notably the rib eye area. Studies suggest that the rib eye area is significantly associated with the carcass lean meat yield (Anderson et al., 2015). Among many carcass traits, the first to be assessed using ultrasonic scanning was the rib eye area, which revealed the importance attached by consumers and farmers to this trait (Arias et al., 2007). Therefore, adding the rib eye area to breeding programs is an important tool to improve sheep carcass values. Hu sheep predominate among Chinese mutton sheep breeds, but show huge variation in their rib eye area because of the selection pressure resulting from breeding methods and geographical differences. The ability to accurately predict the size of the rib eye area early in the life of Hu sheep is extremely valuable for producers to meet the requirements of their target market, and for genetic selection.

In the last 20 years, the heritability of sheep carcass traits has been estimated in many sheep breeds from different regions and countries using different models and approaches (Kefelegn et al., 1999; Greeff et al., 2015; Mortimer et al., 2017; Blasco et al., 2019; Savoia et al., 2019). Several studies have quantified the heritability of certain measured or scored meat quality traits in sheep breeds in different farming and market systems, which demonstrated the existence of genetic variability among these traits, which could be exploited for genetic improvement. However, because the techniques used to obtain phenotypes at the population level are expensive and laborious, it is very rare for measurements of the rib eye area to be used directly in the selection of specialized breeds, e.g., in cattle (Savoia et al., 2019). Carcass merit traits are expressed at the later stages of animal production and are mostly assessed at slaughter, which sacrifices potential breeding stock, although real-time ultrasound imaging technologies can be used to measure the rib eye area (Meirelles et al., 2011; Li et al., 2018).

Reducing the generation interval while maintaining a good level of selection accuracy would improve breeding efficiency (Kefelegn et al., 1999). Early selection is a practical and effective method, which is mainly caried out using genomic selection and molecular marker-assisted breeding (Dekkers, 2003; Tong and Sun, 2015). Identification of the quantitative trait major genes of the rib eye area is necessary. Mutation sites identified using Whole genome sequencing (WGS) represent the ideal pan DNA markers for genetic analyses, because theoretically, they contain all causative polymorphisms. In cattle breeding, genome-wide association studies (GWAs) on production, meat quality, and lactation traits have been reported widely and have been applied in practical production (Lin et al., 2020; Mukiibi, 2020). However, in sheep, only a few GWAs for body size, body weight, and wool traits have been reported (Kominakis and Hager-Theodorides, 2017; Palombo et al., 2020; Tao et al., 2020), and most research used single nucleotide polymorphism (SNP) chips, not the whole genome sequence.

Designing effective breeding programs based on accurate estimates of genetic parameters and molecular markers is one way to solve the problem of slow genetic progression of the rib eye area of sheep. In the present study, the genetic parameters of the rib eye area were estimated using different models, and some genetic correlations between the rib eye area and economic traits were estimated. WGS of High and Low rib eye area groups from a reference population of Hu sheep was performed. The aim was to estimate the heritability of the rib eye area. In addition, we aimed to establish the feasibility of adding the rib eye area trait to breeding goal, and to identify candidate genes that could be used in molecular marker assisted breeding, which will increase the breeding value of Hu sheep.

## Materials and Methods

### Animal Management and Data Collection

In the present study, a total of 1050 male Hu lambs, born between 2018 and 2019, were included. Among them, 776 individuals possessed a complete pedigree record. The full pedigree of the experiment population comprised 1500 individuals, including 70 male parents and 600 female parents, which were from three National Core Breeding Farms of Sheep and Goats (NCBFSG) (Gansu Zhongtian Sheep Industry Co. Ltd., Gansu Zhongsheng Huamei Sheep Industry Co. Ltd., and Gansu Pukang Sheep Industry Co. Ltd.) and a large scale Hu sheep farm (Gansu Sanyang Sheep Industry Co. Ltd.). The population with pedigree information could be used to estimate genetic parameters. Each lamb was weaned at 56 days old and transferred to Minqin Defu Agricultural Technology Co., Ltd. (Performance Measurement Centre, PMC; Gansu Province, China). There were two batches in 2018 (May to September and September to December, respectively) and two batches in 2019 (May to September and September to December, respectively).

From 64 days old, all male Hu lambs were raised indoors in individual 0.8 × 1 m pens until the lambs were 180 days old, and each column of pens had a separate trough and drinking bowl for the sheep to feed freely. Before the study, a 14-day transition period was implemented, during which the proportion of pellets in the diet was gradually increased by 7.1% per day, until the diet became totally granulated. The formula, raw materials, and manufacturing plant of the feed were consistent. The experiment began with lambs at 80 days of age. They were weighed at 80 days to obtain their initial weight (BW80) and raised for up to 180 days. The body weight of each lamb at 180 days old was measured as the final weight (BW180). The feed intake of the lambs during the experimental period was recorded every 20 days. The feed intake for 80–180 days was calculated from five records. Then, we calculated the average daily gain (ADG) and average daily feed intake (ADFI). In addition, individual feed efficiency [residual feed intake (RFI) and feed conversion ratio (FCR)] were estimated. RFI was the residual of the multiple linear regression of ADFI on ADG and the medium metabolic weight [MBW = $\;{(0.5\;\times \left(\text{BW}80+\text{BW}180\right))}^{0.75}$ ] (Tortereau et al., 2020). The FCR was calculated as the ratio of ADFI and ADG.

At the 180 days old, blood was collected and the lambs were slaughtered in accordance with established national standards by Ministry of Agriculture and Rural Affairs of the People’s Republic of China (NY/T 3471-2019). The blood was stored at −20°C until DNA isolation. Twenty-four hours after slaughter, the rib eye muscle area was covered with oleic acid paper from between the fifth and sixth thoracic vertebrae, and used to sketch out the muscle area in a one-to-one ratio. These sketches were scanned using an EPSON Scanner (V19, IDN, Epson, Nagano, Japan) and analyzed using ImageJ (1.8.0; NIH, Bethesda, MD, USA) from which we obtained the rib eye area [REA ( ${\text{cm}}^{2}$ )]. Thickness (cm) of the backfat (BF) above the REA was measured using calipers. We then selected 5% of the lambs (776 × 0.05 ≈  40 individuals) with extreme REA phenotypes and categorized them into high and low groups, respectively.

### DNA Isolation and Sequencing

Genomic DNA of every lamb was isolated using an EasyPure Blood Genomic DNA Kit (TransGen Biotech, Beijing, China), according to the manufacturer’s product description. The quality and integrity of the DNA was measured using the A260/280 ratio and by agarose gel electrophoresis. Then, 1.5 µg of high quality genomic DNA from the high and low groups (80 individuals) were used to generate 80 paired-end sequencing libraries with an inset size of 500 bp. The libraries were sequenced as 150 bp paired end reads using the Illumina NovaSeq PE 150 platform (Illumina, San Diego, CA, USA).

### Sequence Alignment and Single Nucleotide Polymorphism Calling

Quality control was performed on the raw data to improve subsequent analyses using FastQC (Version 0.11.1). Reads with joint sequences and sequences for which the number of undetected bases in single-end sequencing exceeded 10% of the total length of the sequence were eliminated. Sequences with low-quality bases with a mass value of Q ≤ 5 in the single-end sequence were removed. The clean reads were mapped onto the *Ovis aries* reference genome (Oar_rambouillet_v1.0) using BWA (Burrows-Wheeler Aligner) (Version 0.7.8) software with default parameters (Li and Durbin, 2009). To reduce mismatches generated by PCR amplification before sequencing, duplicated reads were removed using Genome Analysis Toolkit (GATK, version 3.4.0) and GATK was used to generate variant call format (VCF) files (Mckenna et al., 2010). SNPs were filtered using Vcftools (version 0.1.14) software and processed as follows: SNPs with call rates <75%, minor allele frequencies (MAF) < 0.05, and minor coverage depth <3. Thus, 80 individuals with 9,875,037 SNPs were kept for further genomic analysis (Danecek et al., 2011).

### Statistical Analysis

#### Estimation of (co)variance Components and Genetic Parameters

To accurately estimate (co)variance components, we constructed an animal mixed model with univariate and bivariate analysis between each pair of feature pairs, using the restricted maximum likelihood (REML) method implemented in the ASReml software (Gilmour et al., 2009). First, the random effects included in the analyses were the direct additive genetic effect of the animal, maternal genetic effects, and random errors. The fixed effects tested and their levels were: Litter size at individual birth (four levels), fattening season (two levels), and the farm before weaning (four levels). All the fixed effects that significantly affected the trait (*p*-value < 0.05), as determined using the ASReml Wald program, were incorporated into the (co)variance estimation. Three models that accounted for the genetic effects and bivariate genetic (co)covariance were fitted and were as follows:

$$Y=\mathrm{X\beta}+Z_{a}a+e$$ (1)

$$Y=\mathrm{X\beta}+Z_{a}a+Z_{m}m_{a}+e\left(\mathrm{cov}\left(a,m_{a}\right)=0\right)$$ (2)

$$G=\left[\begin{array}{cc} A\sigma^{2} & 0\\ 0 & A\sigma_{m}^{2} \end{array}\right]$$

Where Y is the phenotype measurement vector of a trait, while β , a , $m_{a}$ , and e are vectors of fixed, additive direct animal genetic effects, maternal genetic effects, and residual effects, respectively, with association matrices X , $Z_{a}$ , and $Z_{m}$ . G is the variance structure of the model; A is the additive relationship matrix based on the pedigree; and $\;\sigma^{2}$ and $\sigma_{m}^{2}$ are the additive direct and maternal genetic. Residuals were assumed to follow a normal distribution, e∼N(0,R⊗I) .

The bivariate model was as follows:

$$\left[\begin{array}{c} y_{1}\\ y_{2} \end{array}\right]=\left[\begin{array}{cc} X_{1} & 0\\ 0 & X_{2} \end{array}\right]\left[\begin{array}{c} b_{1}\\ b_{2} \end{array}\right]+\left[\begin{array}{cc} Z_{1} & 0\\ 0 & Z_{2} \end{array}\right]\left[\begin{array}{c} a_{1}\\ a_{2} \end{array}\right]+\left[\begin{array}{c} e_{1}\\ e_{2} \end{array}\right]$$ (3)

$$\text{G}=\left[\begin{array}{cc} \sigma_{a_{1}}^{2} & r_{g}\sigma_{a_{1}}\sigma_{a_{2}}\\ r_{g}\sigma_{a_{1}}\sigma_{a_{2}} & \sigma_{a_{2}}^{2} \end{array}\right]⊗A$$

Where y_1_ and y_2_ are the phenotypic measurement vectors of traits 1 and 2, b_1_ and b_2_ are the fixed effect vectors previously described; $a_{1}$ and $a_{2}$ are the vectors of the animal random genetic effects; and e_1_ and e_2_ are the vectors of random residuals. X_1_ and X_2_ are design matrices of the fixed effects and Z_1_ and Z_2_ are design matrices relating traits to random animal genetic effects. $\sigma_{a_{1}}$ and $\;\sigma_{a_{2}}$ represent the additive genetic variances of traits 1 and 2, and rg represents the genetic correlation between them. We used Akaike Information Criterion (AIC), Bayesian Information Criterion (BIC), and Loglik to judge the merits of the model through ASreml software.

#### Candidate Gene Screening

Three methods (fixation index (FST), Fisher’s exact test, and the Chi-squared test) were used to screen mutation sites with significant differences in their allele frequency between the High and Low groups. FST quantified the allele frequency differences between the High and Low groups using Vcftools software (version 0.1.14) (Danecek et al., 2011). We used FST because Weir and Cockerham proposed that genetic structures (or genetic polymorphisms) cause the degree of population differentiation and formulated the FST value (0–1) to represent the degree of allele frequency differences (Cockerham, 1984; Danecek et al., 2011). In the present study, mutation sites with the top 20 FST values were identified as putative selection sites. The genomic data of 80 individuals were subjected to principal components analysis (PCA) [using PLINK (version 1.9)] and permutational multivariate analysis of variance (PERMANOVA) (using Vegan R packages) to verify whether there is population stratification. We constructed a genome-wide case/control design within the High and Low groups using Fisher’s exact test and the Chi-squared test to calculate the *p*-values using PLINK (version 1.9) (Purcell et al., 2007). In addition, the first five principal components were added as covariates for the association analysis using Fisher’s test. We used the false discovery rate (FDR) method to set the *p*-value threshold. The genome-wide threshold value was calculated according to an FDR of 0.05. The p.adjust function of R (version 4.0.3) was used to calculate the FDR. (Supplementary Tables S1, S2).

#### Genotyping and Association

We selected SNPs that met the requirements in all three screening methods (FST, Fisher’s exact test, and the Chi-squared test). PCR extension primers for the SNPs were designed using the genomic DNA sequences. DNA samples from 1050 lambs with phenotypic records were genetyped using KAspar genotyping technology (LGC Genomics, Hoddesdon, UK) (Smith and Maughan, 2015) to further screen the SNPs. The general linear model analysis in the SPSS 22.0 software (IBM Corp., Armonk, NY, USA) was used to analyze the association between the genotypes and the REA (Zhang et al., 2019). The linear model with the fixed and covariate effects was as follows:

$$y_{\mathrm{igkl}}=\mu +\mathrm{Genotyp}e_{i}+\mathrm{Batc}h_{j}+\mathrm{Seaso}n_{k}+180\mathrm{BW}+\varepsilon_{\mathrm{igkl}}$$

In this model, ${\text{y}}_{igkl}$ is the vector of the phenotypic information; μ is the population mean; ${\text{Genotype}}_{i}$ is the $i^{th}$ genotype; ${\text{Batch}}_{j}$ is the effect of the $j^{th}$ batch; $Season_{k}$ is the effect of the $k^{th}$ season; 180BW was a covariate added to the model; $\varepsilon_{igkl}$ is the residual corresponding to the trait observation value with $\varepsilon ∼\text{N}\left(0,\sigma^{2}\right)$ . All effects met the statistically significant criterion (*p* < 0.05).

## Results

### Phenotype and Genetic Parameters

**FIGURE 1:**
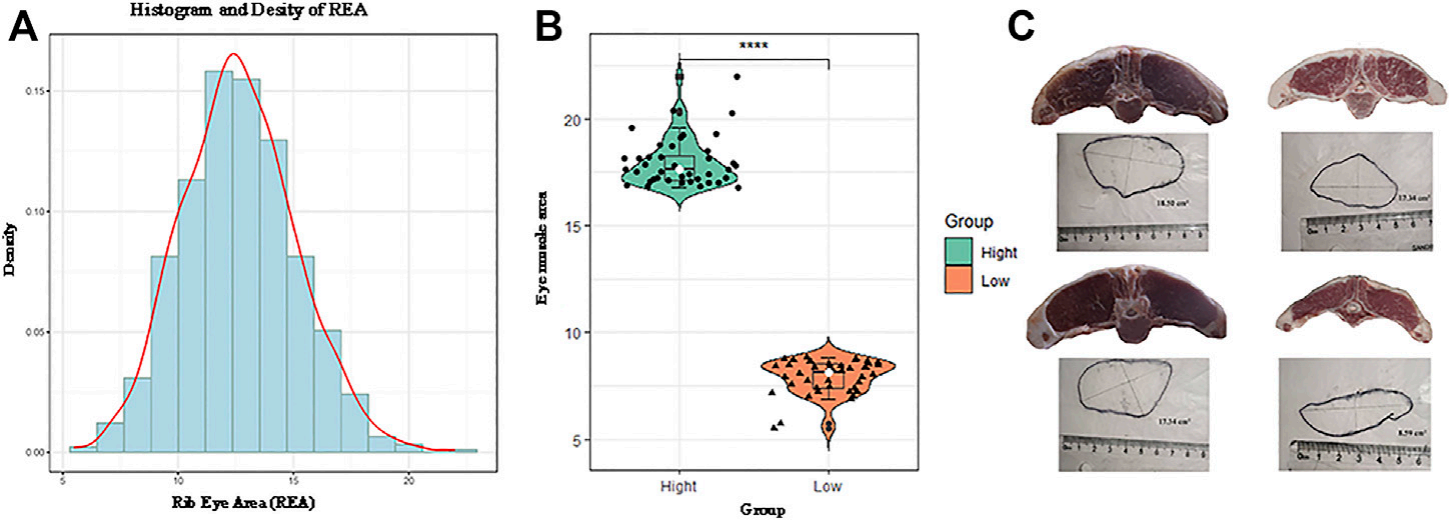
Probability density map of the original phenotype data **(A)**. The distribution of the included rib eye area (REA) from the high and low groups **(B)**. Photograph of the REA **(C)**.

In the present study, 776 animals with their REA phenotypes were included to estimate the genetic parameters. The average REA was 12.64 ± 2.46 ${\text{cm}}^{2}$ . The probability density of the phenotype data is shown in Figure 1A. The REA of the high (mean = 17.97 ± 1.14 ${\text{cm}}^{2}$ ) and low (mean = 7.89 ± 0.79 ${\text{cm}}^{2}$ ) groups screened from the large population are shown in Figure 1B.

Heritability and variance components for the REA are shown in Table 1. The REA heritability estimated using different models showed little difference (0.32 and 0.28). The litter common environmental effect accounted for the majority of the REA. The REA trait showed moderate heritability. Maternal heritability was significantly greater than zero for the REA trait in Hu sheep; however, the influence of the maternal genetic effect on the heritability and the additive variance component estimation of the REA trait was minimal. We calculated the parameters (AIC and BIC) of the two evaluation models (Table 1).

**TABLE 1:** *Estimates of heritability (h2) and variance components*.

	log	$\sigma_{a}^{2}$	$\sigma_{e}^{2}$	$\sigma_{m}^{2}$	$h^{2}$	$h_{m}$	AIC	BIC
Mod1	−1357.78	1.42 ± 0.62	3.04 ± 0.58		0.32 ± 0.13		2719.568	2729.525
Mod2	−1356.14	1.27 ± 0.83	2.96 ± 0.61	0.25 ± 0.43	0.28 ± 0.13	0.06 ± 0.05	2718.275	2733.210

Note: Mod1 $Y=X\beta +Z_{a}a+e$ ; Mod2 $Y=X\beta +Z_{a}a+Z_{m}m_{a}+e$ ; ${\text{σ}}_{\text{a}}^{2}$ additive effect variance component; ${\text{σ}}_{\text{e}}^{2}$ residual variance component; ${\text{σ}}_{\text{m}}^{2}$ Maternal genetic effect variance component; ${\text{h}}^{2}$ heritability; ${\text{h}}_{\text{m}}$ Maternal heritability; AIC: Akaike information criterion; BIC: Bayesian information criterion.

Estimates of genetic and phenotype correlations between the REA and important economic traits (body weight, feed intake, feed efficiency, and backfat, respectively) are shown in Table 2. The correlations were high for the REA and production traits, with high genetic correlations between BW80, BW180, ADFI80–180, ADG80–180, and REA (0.58, 0.80, 0.55, and 0.91, respectively). Positive moderate phenotype correlations were estimated between the REA and production traits, at 0.32, 0.42, 0.37, and 0.34, respectively. Genetic correlations between feed efficiency traits and the REA were negligible (−0.05 and −0.01). Low correlations were estimated between feed efficiency and the REA at the phenotypic level (−0.02 and 0). BF and the REA were genetically correlated with a large standard error, while there was a positive phenotypic correlation between the BF and the REA.

**TABLE 2:** *Estimates of genetic (rg) and phenotypic (rp) correlations for rib eye area, carcass, and important economic traits in Male Hu sheep*.

Character	REA	
	rg	rp
BW80	0.58 ± 0.18	0.32 ± 0.03
BW180	0.80 ± 0.19	0.42 ± 0.03
ADFI80–180	0.55 ± 0.24	0.37 ± 0.03
ADG80–180	0.91 ± 0.16	0.34 ± 0.03
FCR80–180	−0.05 ± 0.08	-0.02 ± 0.03
RFI80–180	−0.01 ± 0.10	0 ± 0.03
BF	0.06 ± 0.33	0.11 ± 0.03

Note: rg : genetic correlation; rp: phenotypic correlation; REA = rib eye area (cm2); BW80 = weight at 80 days old (kg); BW180 = weight at 180 days old (kg); ADFI = average daily feed intake (kg); ADG80–180 = average daily gain (kg); FCR80–180 = Feed conversion ratio; RFI80–180 = residual feed intake (kg); BF = backfat (cm).

### Candidate Genes and SNPs

To identify variation in the REA in sheep genomes, we identified the candidate genes associated with an increased REA in Hu sheep. We performed WGS of Hu lambs in the high and low groups. We obtained a total of 700 Gb of raw data, with an average depth of 5-fold for each individual. The average number of reads was 58785931 and the average %GC was 43.6. (Supplementary Table S3). The effective sequencing rate was 99.42%, which suggested that the data had high quality and could be used for further in-depth analysis.

**FIGURE 2:**
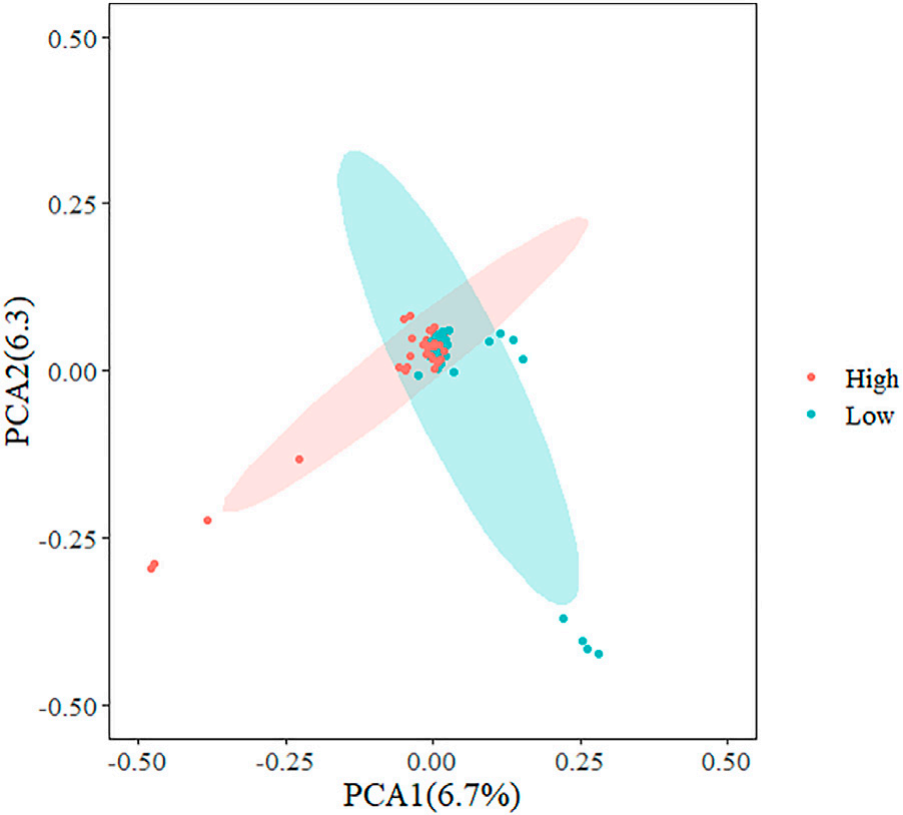
Principal component analysis (PCA) plots of Hu sheep whole genome sequencing data.

**FIGURE 3:**
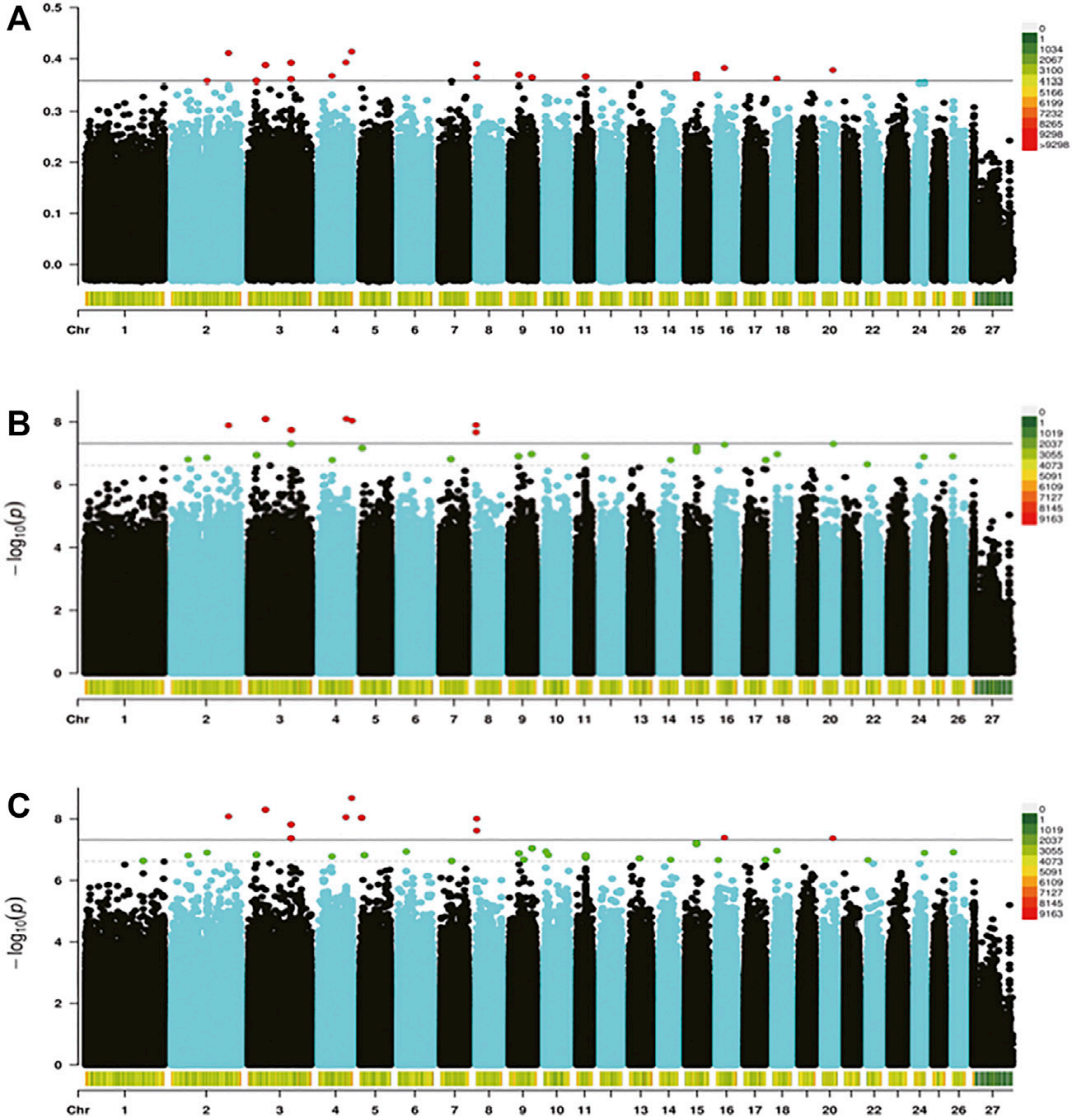
Genome-wide distribution of Fst **(A)**, Fisher’s exact test **(B)**, and the chi-squared test **(C)**. The horizontal black line in the figure shows the threshold of methods at the secondary identified signal [-log (4.82 $\times 10^{-8})$ and -log (3.77 $\times 10^{-8}$ ), respectively].

In the present study, three methods were used to identify SNPs and genes associated with the REA in Hu sheep. Figure 2 shows that population stratification did not appear. The top two principal components accounted for a small proportion of the variance (6.7 and 6.3%). In addition, we performed permutational multivariate analysis of variance (PERMANOVA) using vegan packages, which produced a *p*-value of 0.0014. This verified that there were no subgroups in the population (*p* < 0.05). To detect strongly selected signals, we searched the sheep genome for single SNPs with an increased genetic distance (FST). We set a threshold to select the top 20 SNPs and we used the PLINK software for PCA. The first five principal components were included in Fisher’s exact test. According to the *p*-value of every SNP, eleven significant SNPs (Supplementary Table S1) were identified for the REA at the secondary identified signal (*p* < 4.82 $\times 10^{-8}$ ). Then, we used the Chi-squared test to detect seven significant SNPs (Supplementary Table S2) for the REA at the secondary identified signal (*p* < 3.77 $\times 10^{-8}$ ). Manhattan maps of the GWAS are shown in Figure 3.

We found that the SNPs selected using the three methods were decreasing and coincident (Fst > Fisher’s exact > Chi-squared). Subsequently, seven target SNPs were selected that overlapped among the three methods and were annotated to the closest gene in the Oar_rambouillet_v1.0 genome. Six genes containing these seven SNPs were defined as candidate genes (Table 3).

**TABLE 3:** *The significant SNPs for the rib eye area in Hu sheep*.

Chr	Position	Gene	Fst-value	Fisher’s- *p*-Value	Chi-squared- *p*-Value	REF	ALT		Post type
chr2	218145714	*ALS2*	0.41	8.38e-09	1.28e-08	T	C		downstream
chr3	65927208	*ST6GAL2*	0.39	5.04e-09	8.02e-09	T	C		downstream
chr3	163720238	*LOC105611989*	0.39	1.52e-08	1.81e-08	A	T		downstream
chr4	104628299	*PLXNA4*	0.39	8.81e-09	7.92e-09	TC	T		intronic
chr4	126636893	*DPP6*	0.41	2.10e-09	9.13e-09	A	G		intronic
chr8	2261361	*COL12A1*	0.39	9.38e-09	1.26e-08	T	A		intronic
chr8	2261369	*COL12A1*	0.36	2.39e-08	2.14e-08	T	A		intronic

Gene: Gene obtained by annotation of significant SNP.

**TABLE 4:** *Association between the REA and different genotypes of the ST6GAL2, LOC105611989*, *DPP6*, *and COL12A1 genes*.

Gene/Loci	Genotype	N	*p*-value	REA	HWE (*p*-value)
*ST6GAL2*	T:T	93	0.40	12.42 ± 2.18	<0.001
chr3:65927208	T:C	356		12.68 ± 2.33	
T > C	C:C	587		12.38 ± 2.37	
*LOC105611989*	A:A	145	0.0004	11.78 ± 2.43^c^	0.792
chr3:163720238	A; T	479		12.40 ± 2.35^b^	
A > T	T:T	415		12.82 ± 2.27^a^	
*DPP6*	A:A	25	0.019	12.89 ± 2.30^a^	0.007
chr4:126636893	A:G	198		12.67 ± 2.27^a^	
A > G	G:G	727		12.27 ± 2.29^b^	
*COL12A1*	T:T	222	<0.01	12.50 ± 4.46^a^	0.094
chr8:2261361	A:T	449		12.44 ± 2.22^ab^	
T > A	A:A	279		12.13 ± 2.21^b^	
*COL12A1*	T:T	267	0.19	12.12 ± 2.22^b^	0.248
chr8:2261369	A:T	460		12.47 ± 2.25^a^	
T > A	A:A	226		12.43 ± 2.22^ab^	

*p*-value: Significance of genotype as fixed effect in linearity model (*p* < 0.05); REA: The mean value of rib eye muscle area in three genotypes; The letters (a,b,c) represents the mean value with different superscripts differ significantly (*p* < 0.05); HWE(*p*-value): The Significance of Hardy–Weinberg equilibrium.

### Association Analysis of the Candidate Genes With the REA

**FIGURE 4:**
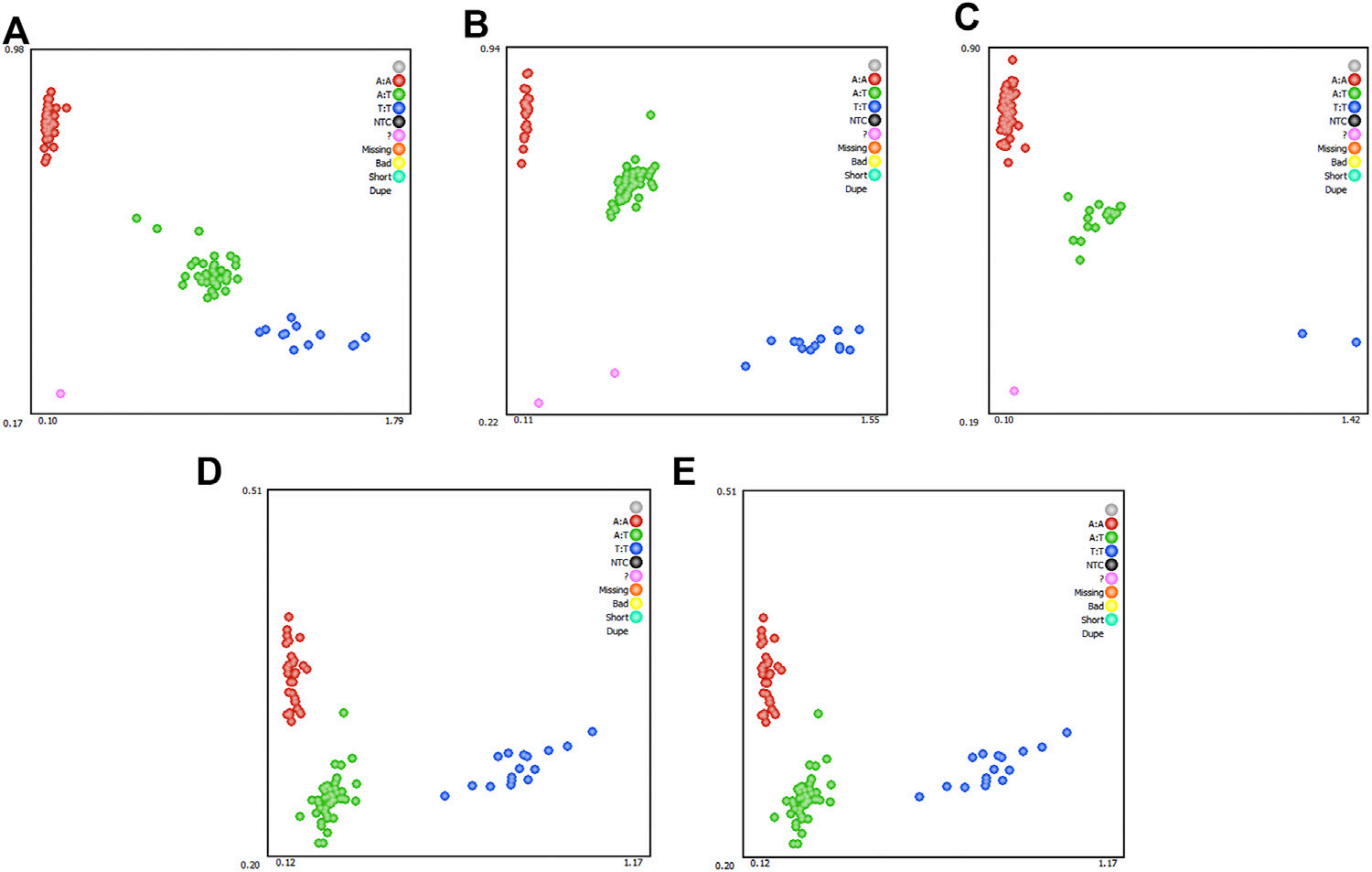
Genotyping of *ST6GAL2* **(A)**, *LOC105611989* **(B)**, *DPP6* **(C)**, and *COL12A1* **(D and E)** single nucleotide polymorphisms (SNPs). Note: Red, green, and blue represents three genotypes, respectively; while the pink dots indicate genotyping failure.

To determine whether the selected candidate genes had a significant effect in the REA, in the enlarged experimental population (n = 1050), five SNPs (*ST6GAL2* (encoding ST6 beta-galactoside alpha-2,6-sialyltransferase 2) SNP g.65927208 T > C, *LOC105611989* SNP g.65927208A > T, *DPP6* (encoding dipeptidyl peptidase like 6) SNP g.126636893A > G, *COL12A1* (encoding collagen type XII alpha 1 chain) SNP g.2261361 T > A, and *COL12A1* SNP g.2261369 T > A) were subjected to genotyping, which generated three genotypes (Figure 4). The result of association analysis indicated that *LOC105611989* SNP g.65927208A > T, *DPP6* g.126636893 SNP A > G, *COL12A1* SNP g.2261361 T > A, and *COL12A1* SNP g.2261369 T > A were significantly associated with the REA (*p* < 0.05); however, *ST6GAL2* SNP g.65927208 T > C did not have a significant impact on the REA. For *LOC105611989*, the male Hu sheep with the TT genotype had largest REA and those with the AA genotype had the smallest REA among the three genotypes (*p* < 0.05). The REA of male Hu sheep carrying the AA and AG genotypes of the *DPP6* SNP g.126636893A > G was increased compared with that in male Hu sheep carrying the GG genotype (*p* < 0.05). The effect of the *COL12A1* g.2261361 T > A and *COL12A1* g.2261369 T > A SNPs s were significant on the REA. For *COL12A1* SNP g.2261361 T > A, male Hu sheep with the TT genotype had a larger REA than those with the AA genotype. By contrast, the REA in the experimental population with the TT genotype of the *COL12A1* g.2261369 T > A SNP was significantly lower compared with that in the sheep with the AT genotype.

*LOC105611989* SNP g.65927208A > T, *COL12A1* SNP g.2261361 T > A, and *COL12A1* SNP g.2261369 T > A were in Hardy–Weinberg equilibrium (*p* > 0.05). However, *ST6GAL2* SNP g.65927208 T > C and *DPP6* SNP g.126636893A > G did not conform to the Hardy–Weinberg equilibrium (*p* < 0.05).

## Discussion

Genetic improvement has played an important role in productivity gains in animal farming. The REA is a valuable economic trait that could affect production traits in animals (Savoia et al., 2019). The REA has been reported in many studies that mainly investigated nutrition (Silva et al., 2019; Jiao et al., 2020; Redoy et al., 2020); however, there have been relatively few studies reporting the genetic mechanism of the REA, and it was not until 2013 that a GWAS for the REA in sheep was reported (Zhang et al., 2013), although GWAS studies had been used earlier to study the REA in other livestock.

In our study, we estimated the genetic parameters of the REA in Hu breeding, and candidate gene mining was carried out. The heritability of the REA-based genetic parameters with different models was moderate. Roden et al. reported lower estimates of for rib eye muscle depth and width (0.11 and 0.08) in Scottish Blackface breed than those in the present study (Roden et al., 2003). In our population, maternal genetics was not an important influence on the REA. Previous studies on different cattle breeds (Nellore, Red Angus, and Piedmontese) reported $h^{2}$ values for the REA ranging from 0.21 to 0.26 (Boldt et al., 2018; Savoia et al., 2019). In 2007, rib eye muscle depth heritability of Kivircik lambs was estimated using Bayesian inference as 0.23 (Cemal et al., 2016). Our results of the heritability of the REA are also similar to those of a previous report (0.23) (Pollott and Greeff, 2004).

In addition, we found interesting correlations between the REA and certain important economic traits. Positive genetic correlations were estimated between the REA and production traits (0.58 for BW80, 0.80 for BW180, 0.55 for ADFI, and 0.91 for ADG). As expected, the size of an individual has a decisive impact on the size of the REA. Mortimer et al. reported high genetic correlations (from 0.37 to 0.70) between the REA and body weight at different stages (Mortimer et al., 2018). Phenotype correlation analysis supported the results of the genetic correlation analysis (Table 2).

We identified negligible genetic correlations between REA and feed efficiency traits (−0.05 and −0.01), and ADFI and BW correlated positively with the REA, suggesting that animals with a larger REA grow faster and consume more feed, so it is expected that their feed efficiency would be the same. A negative genetic correlation between the measured muscle depth and feed efficiency traits (−0.3 ± 0.15) with RFI and −0.15 ± 0.18 with FCR) has been observed in other breeds (Tortereau et al., 2020). This correlation suggests that animals with a larger REA are expected to have excellent feed efficiency. In 2004, the genetic correlation between fat depth and the REA was estimated as 0.05 (Pollott and Greeff, 2004). In our study, negative genetic relationship between the REA and BF was estimated (0.06) However, the genetic correlation between the REA and BF had a large standard error. Fat serves as an energy store in animals. A previous study found evidence that the genetic correlation between fat reserves and production traits can change across environments (Pollott and Greeff, 2004). Based on the results of the genetic parameters, we suggest that the REA trait should be incorporated into breeding of Hu sheep.

The REA is commonly used to reflect the muscular development of the carcass. To further investigate the mechanisms affecting the REA, we identified genes that regulate the REA in the Hu sheep genome using three methods. SNPs were identified in six genes: *ALS2* (encoding Alsin Rho guanine nucleotide exchange factor 2) SNP g.218145714 T > C, *ST6GAL2* SNP g.65927208 T > C, *LOC105611989* SNP g.163720238A > T, *PLXNA4* (encoding plexin A4) SNP g.104628299 TC > T, *DPP6* SNP g.126636893A > G, *COL12A1* SNP g.2261361 T > A, and *COL12A1* SNP g.2261369 T > A.

The mutations in the *ALS2* gene may case Familial amyotrophic lateral sclerosis type 2, which is a juvenile autosomal recessive motor neuron disease. The *ALS2* gene product, ALS2/alsin, forms a homophilic oligomer and acts as a guanine nucleotide–exchange factor (GEF) for the small GTPase Rab5. This oligomerization is crucial for both Rab5 activation and ALS2-mediated endosome fusion and maturation in cells (Asako et al., 2003; Sato et al., 2018). *ALS2* might act as a modulator in neuronal differentiation and development through regulation of membrane dynamics (Otomo et al., 2008). Actin and myoglobulin is involved in guiding regulation of membrane dynamics (Poukkula et al., 2011). Macro pinocytosis is the cytoplasmic membrane folding mediated by actin that can non-selectively encapsulate a large number of extracellular nutrients and liquid macromolecules, and transport them to the lysosome for degradation after the cell is stimulated by nutrient factors or pho wave ester. Macro pinocytosis alters the nutritional physiology of muscle cells. The development and nutritional metabolism of muscle cells are inseparable from this process. The mutation of *ALS2* has led to a certain degree of muscle atrophy in the longissimus dorsi muscle in Hu sheep. This caused the REA to become smaller. The association analysis of the larger group also supported this view.

Studies have reported that *PLAXN4* and Semaphorin3A show a high degree of interaction (Quintá et al., 2014; Poltavski et al., 2019; Limoni et al., 2021). *PLAXN4* is used as a star gene in the Axon guidance - *Homo sapiens* KEGG category. *PLAXN4* acts directly upstream of Rac, p21-activated kinase (PAK), and RhoD functions. Rho GTPases are known to regulate actin dynamics (Ridley, 2006). In motile cells, during the process of mitosis, the activities of GTPases of different Rho families are separated in intracellular space (Pal et al., 2020). A previous study found that RhoD could stimulate actin polymerization and affect plasma membrane protrusion and/or vesicular traffic (Ridley, 2006). Pal et al. (2020) reported that manipulating Rac and Arp2/3 activity resulted in polar body defects during mitosis and meiosis in sea urchin embryos and sea star oocytes, and suggested that Rac and Arp2/3-mediated actin networks might directly antagonize Rho signaling. PAK is an effector downstream target of Rho-GTPases Rac1 and Cdc42, which can activate its downstream target cofilin via LIM kinase-1, and subsequently supports cell migration and invasion through the polymerization of actin filaments (Mierke et al., 2020). The Rac/PAK/GC/cGMP signaling pathway allows the action of RAC and PAK to affect actin simultaneously (Guo et al., 2010). Actin forms part of the muscle cytoskeleton. If there is a lack of actin production, the development and mitosis of the longissimus dorsi muscle will be affected, resulting in muscular dysplasia and thus reducing the rib eye area muscle.

DPP6 is an auxiliary subunit of the Kv4 family of voltage-gated K (+) channels, which is known to enhance channel surface expression and potently accelerate their kinetics. Many studies suggest that *DPP6* is consistently and strongly associated with susceptibility to amyotrophic lateral sclerosis (ALS) in different human populations of European ancestry (Del Bo et al., 2008; Es et al., 2008; Lin et al., 2014). This suggests that *DPP6* acts as a susceptibility gene for muscle atrophy symptoms in humans. It is possible that DPP6 has the same effect on muscle atrophy in animals.

After fitting multiple factors in the association analysis in a large population, we found that the *COL12A* gene is related to the size of the REA of Hu sheep. *COL12A* is mainly involved in the degradation of the extracellular matrix pathway and collagen chain trimerization. In 2014, a myopathy/anhydrotic ectodermal dysplasia (EDA)-like disease caused by heterozygous *COL12A1* variants was reported (Punetha et al., 2017). Mice with *Col12A1* knockout showed muscle weakness with decreased grip strength, combined with bone fragility, short stature, and kyphoscoliosis (Yaqun et al., 2014). Delbaere et al. (2020) believed that mixed myopathy was caused by defects in collagen XII and VI, and by variant-specific alterations in the extracellular matrix resulting from *COL12A1* mutation. We believe that this disease also occurs in Hu sheep.

In addition, we performed genotyping and association analysis on the seven SNPs in the enlarged experimental population. Five loci were typed successfully and three loci were significantly associated with the REA. These three SNPs could be used for marker-assisted selection. Therefore, we hypothesized that these candidate genes might affect muscle development in Hu sheep. We cannot perform whole genome sequencing in a large Hu sheep population because of the cost; however, further studies are needed to confirm the biological effects of the identified SNPs in the rib eye area.

According to the association analysis of candidate genes, we found that in male Hu sheep *ST6GAL2* SNP g. 65927208 T > C has no association with the REA. The results showed that the SNP has skewness in this population, which led to a lack of association with the REA. Although this research verified the association between candidate SNPs and the REA in a large population, verification at the cell and protein levels is still needed.

## Conclusion

The results obtained in the present study showed that the rib eye area was heritable and there is genetic variability, which could theoretically be exploited for the genetic improvement of sheep. We identified *LOC105611989* SNP g.65927208A > T, *DPP6* SNP g.126636893A > G, *COL12A1* SNP g.2261361 T > A, and *COL12A1* SNP g.2261369 T > A, whose protein products have metabolic functions in muscle cells, as candidate genetic markers of the rib eye area. Our results could be applied to marker assisted breeding of sheep and are expected to improve for the slow genetic progress of the rib eye area in sheep. Moreover, this study provides important information for estimating the heritability parameters of the rib eye area and for molecular breeding of carcass traits in Hu sheep.

## Data Availability

The datasets presented in this study can be found in online repositories. The names of the repository/repositories and accession number(s) can be found below: https://www.ncbi.nlm.nih.gov/search/all/?term=PRJNA779188.
