# Supplementary material for: Whole Genome Sequencing Analysis to Identify Candidate Genes Associated With the rib eye Muscle Area in Hu Sheep
Source: Front Genet. 2022 Mar 14;13:824742. doi: 10.3389/fgene.2022.824742 (PMC8964300; doi:10.3389/fgene.2022.824742)
Supplement: Supplementary file 1 [file DataSheet2.docx]

**Table S1** *The significant mutation site of Fisher’s exact test with FDR Rectified (p < 0.05).*

| Chr | Pos | REF | ALT | *p*-value | *p*-value (FDR) |
| --- | --- | --- | --- | --- | --- |
| 2 | 2:218145714 | C | T | 8.35E-09 | 0.0161786 |
| 3 | 3:65927208 | T | C | 5.04E-09 | 0.0161786 |
| 3 | 3:163720238 | A | T | 1.52E-08 | 0.0214853 |
| 3 | 3:163736508 | G | T | 4.27E-08 | 0.0383062 |
| 4 | 4:104628299 | TC | T | 8.81E-09 | 0.0161786 |
| 4 | 4:126636893 | A | G | 2.10E-09 | 0.0161786 |
| 5 | 5:6750184 | G | T | 9.16E-09 | 0.0161786 |
| 8 | 8:2261361 | A | T | 9.83E-09 | 0.0161786 |
| 8 | 8:2261369 | A | T | 2.39E-08 | 0.0295387 |
| 16 | 16:31435355 | A | G | 4.06E-08 | 0.0383062 |
| 20 | 20:39814866 | GT | G | 4.26E-08 | 0.0383062 |

Note:REF: reference site genotype; ALT: Mutation site genotype ; *p*-value (FDR): FDR Rectified *p*-vaule

**Table S2** *The significant mutation site of Chi-squared test with FDR Rectified (p < 0.05).*

| Chr | Pos | REF | ALT | *p*-value | *p*-value (FDR) |
| --- | --- | --- | --- | --- | --- |
| 2 | 2:218145714 | C | T | 1.28E-08 | 0.0253 |
| 3 | 3:65927208 | T | C | 8.02E-09 | 0.0253 |
| 3 | 3:163720238 | A | T | 1.81E-08 | 0.02974 |
| 4 | 4:104628299 | TC | T | 7.92E-09 | 0.0253 |
| 4 | 4:126636893 | A | G | 9.13E-09 | 0.0253 |
| 8 | 8:2261361 | A | T | 1.26E-08 | 0.0253 |
| 8 | 8:2261369 | A | T | 2.14E-08 | 0.03013 |

Note:REF: reference site genotype; ALT: Mutation site genotype; *p*-value (FDR): FDR Rectified *p*-vaule
